# Blast-Related Mild TBI Alters Anxiety-Like Behavior and Transcriptional Signatures in the Rat Amygdala

**DOI:** 10.3389/fnbeh.2020.00160

**Published:** 2020-09-30

**Authors:** Jennifer Blaze, Inbae Choi, Zhaoyu Wang, Michelle Umali, Natalia Mendelev, Anna E. Tschiffely, Stephen T. Ahlers, Gregory A. Elder, Yongchao Ge, Fatemeh Haghighi

**Affiliations:** ^1^Department of Neuroscience, Icahn School of Medicine at Mount Sinai, New York, NY, United States; ^2^Friedman Brain Institute, Icahn School of Medicine at Mount Sinai, New York, NY, United States; ^3^Research and Development Service, James J. Peters Veterans Affairs Medical Center, Bronx, NY, United States; ^4^Department of Neurotrauma, Operational and Undersea Medicine Directorate, Naval Medical Research Center, Silver Spring, MD, United States; ^5^Department of Psychiatry, Icahn School of Medicine at Mount Sinai, New York, NY, United States; ^6^Department of Neurology, Icahn School of Medicine at Mount Sinai, New York, NY, United States; ^7^Neurology Service, James J. Peters Veterans Affairs Medical Center, Bronx, NY, United States

**Keywords:** mTBI, amygdala, blast, anxiety, transcriptome

## Abstract

The short and long-term neurological and psychological consequences of traumatic brain injury (TBI), and especially mild TBI (mTBI) are of immense interest to the Veteran community. mTBI is a common and detrimental result of combat exposure and results in various deleterious outcomes, including mood and anxiety disorders, cognitive deficits, and post-traumatic stress disorder (PTSD). In the current study, we aimed to further define the behavioral and molecular effects of blast-related mTBI using a well-established (3 × 75 kPa, one per day on three consecutive days) repeated blast overpressure (rBOP) model in rats. We exposed adult male rats to the rBOP procedure and conducted behavioral tests for anxiety and fear conditioning at 1–1.5 months (sub-acute) or 12–13 months (chronic) following blast exposure. We also used next-generation sequencing to measure transcriptome-wide gene expression in the amygdala of sham and blast-exposed animals at the sub-acute and chronic time points. Results showed that blast-exposed animals exhibited an anxiety-like phenotype at the sub-acute timepoint but this phenotype was diminished by the chronic time point. Conversely, gene expression analysis at both sub-acute and chronic timepoints demonstrated a large treatment by timepoint interaction such that the most differentially expressed genes were present in the blast-exposed animals at the chronic time point, which also corresponded to a *Bdnf*-centric gene network. Overall, the current study identified changes in the amygdalar transcriptome and anxiety-related phenotypic outcomes dependent on both blast exposure and aging, which may play a role in the long-term pathological consequences of mTBI.

## Introduction

Traumatic brain injury (TBI) affects 1.7 million Americans each year according to the Centers for Disease Control and Prevention (Faul et al., 2010). Between 2000 and 2019 over 300,000 members of the United States Armed Forces were diagnosed with TBI, and of these 82.8% were diagnosed with mild TBI (mTBI; Defense and Veterans Brain Injury Center, 2019). It has also been suggested that these injury rates are likely to be underestimated (Chase and Nevin, 2014). The modes of injury that can result in mTBI vary from exposure to improvised explosive devices to closed head injuries. Of particular interest to the Veteran population are the long-term sequelae of mTBI, which can include mood and anxiety disorders, posttraumatic stress disorder, heightened suicidality, and diminished cognitive capacity with deficits in attention and memory. Also, there is a growing concern for the long-term neuropathological consequences following repeated exposure to physically traumatic events such as chronic traumatic encephalopathy (CTE). With such a large number of returning Veterans sustaining TBI, understanding the molecular circuitry involved in the long-term sequalae resulting from TBI is critically important to the health and productivity of our Military and Veteran population.

Various animal models have been established to study TBI-related pathology, including direct exposure to live explosives and controlled blast waves from compressed air generators, with the use of these models increasing in recent years. In particular, our group has developed a model of blast overpressure in rats (Ahlers et al., 2012) to explore the pathology and molecular outcomes resulting from mild blast injury (Elder et al., 2012; Haghighi et al., 2015; Gama Sosa et al., 2019). High-level blast exposure is associated with hemorrhagic lesions as well as histological effects including axonal, glial, microglial, and myelin changes (Elder et al., 2010). Until recently, the animal models have mainly used powerful high-level blast exposures capable of causing extreme intracranial damage, but our collaborative efforts and other groups have now begun to investigate the effects of a lower-intensity blast model known as repeated blast overpressure (rBOP). The mild nature of this animal model seems to more closely resemble human blast exposure in current war zones and operational settings (Moochhala et al., 2004; Saljo et al., 2010; Park et al., 2011; Pun et al., 2011; Rubovitch et al., 2011). Our group previously found that chronic and persistent behavioral effects can be found in rats months after rBOP, including increased anxiety-like behavior consistent with the manifestation of PTSD-like symptoms (Elder et al., 2012).

Behavioral phenotypes following mTBI in both rodents and humans have been associated with changes in gene expression in the brain and periphery (Gill et al., 2017). Modulation of gene expression is one way the brain creates new homeostasis in response to experience, including response to injury, making transcriptomic analyses following mTBI a particularly interesting area of study. As previously described, the advent of animal models of mTBI, such as our rBOP model, has allowed us to query the rodent brain transcriptome following blast injury. Our group previously found changes to neuronal gene expression and DNA methylation in the rat frontal cortex following rBOP. Transcriptional changes are present following blast exposure in the rodent frontal cortex (Hayes et al., 1995; Haghighi et al., 2015) and hippocampus (Phillips and Belardo, 1992; Hayes et al., 1995; Hua Li et al., 2004; Tweedie et al., 2013; Luo et al., 2017), but transcriptional changes in other brain regions important in emotion and learning, such as the amygdala, have not yet been investigated.

The majority of work investigating rodent models of TBI has focused on more sub-acute changes to behavior or structural/functional changes in the brain (days to weeks following blast), but more recent studies from our group suggest that deleterious effects of mTBI are long-lasting or may emerge 8–10 months following the blast exposure (Perez-Garcia et al., 2016; Gama Sosa et al., 2019). The long-term outcomes of mTBI are of particular interest due to the high levels of neurodegenerative disorders in aging, mTBI-exposed Veterans (Elder, 2015).

In the current study, we sought to replicate and extend previous findings (Elder et al., 2012) by determining both the sub-acute and chronic effects of rBOP in a similar rodent model. Specifically, we chose to investigate anxiety-like and cognitive behavior induced by rBOP and aimed to correlate these changes to transcriptional changes in the rodent amygdala, which has been shown to exhibit molecular, functional, and structural alterations following TBI and mTBI in humans (Depue et al., 2014; Han et al., 2015) and animal models (Elder et al., 2012; Heldt et al., 2014; Palmer et al., 2016; Hall et al., 2017; Hubbard et al., 2018). We hypothesized that rBOP would produce increased anxiety-like behavior, enhanced fear learning, and significantly alter gene expression in the amygdala, with the most drastic changes in aged rats that experienced rBOP. Identifying aberrant regulation of gene expression and behavior immediately following rBOP or months following exposure is critical in revealing molecular mechanisms leading to the manifestation of psychopathology, and possibly neuropathology, later in life.

## Materials and Methods

### Subjects

All study procedures were approved by Institutional Animal Care and Use Committees of the Naval Medical Research Center and the James J. Peters Veterans Administration (VA) Medical Center, and were conducted in conformance with Public Health Service policy regarding the humane care and use of laboratory animals and the National Institutes of Health (NIH) Guide for the Care and Use of Laboratory Animals. Specifically, male Long Evans rats weighing between 250–300 grams and approximately 10–12 weeks of age (Charles River Laboratories International, Inc., Wilmington, MA, USA) were utilized as subjects. NMRC’s animal housing rooms were maintained at a temperature between 17.8–26°C, humidity level between 30–70%, and 12:12 light-dark cycle. JJPVAMC’s animal housing rooms were maintained at a temperature between 21–22°C, humidity level between 30–70%, and 12:12 light-dark cycle. In both facilities, animals were individually housed in standard clear plastic cages complete with Bed-O’Cobs (The Andersons, Inc., Maumee, OH, USA) bedding and EnviroDri nesting article (Sheppard Specialty Articles, Milford, NJ, USA) with food and water provided *ad libitum*.

### Repeated Blast Overpressure Exposure (rBOP)

Rats were exposed to multiple BOP events with the Walter Reed Army Institute of Research (WRAIR) shock tube as described previously (Elder et al., 2012). Animals were handled for 2 min a day for the 2 days before the beginning of the blast procedure which itself requires handling for the 3 days of blast exposure. For the rBOP procedure, animal subjects were anesthetized with 5% isoflurane gas for at least 2 min, outfitted with plastic restraint cones, and positioned in the shock tube’s animal holder. Animals were situated prone (dorsal side up), facing the direction of the blast wave emitted by the WRAIR shock tube. The head, upper torso, and lower torso areas of the subjects were further secured with three rubber tourniquets to restrict movement without hindering respiration. Subjects assigned to blast-exposed groups received a single 74.5 kPa (≈10.8 psi) BOP event every 24 h for 72 h. Control subjects were treated identically to blast-exposed subjects barring the BOP exposures. After sham or blast treatment, subjects were placed in boxes and transferred from NMRC to JJPVAMC within 10 days *via* approved commercial transport carrier. Upon arriving at JJPVAMC, the sub-acute cohort of animals was acclimated to the facility for seven days after which they were handled three times a week for 2 weeks before the onset of the behavioral assessment battery. The chronic cohort remained in the JJPVAMC facility for 12–14 months before being handled three times a week for 2 weeks before the onset of the behavioral assessment battery.

### Behavioral Assessment Battery

**Figure 1:**
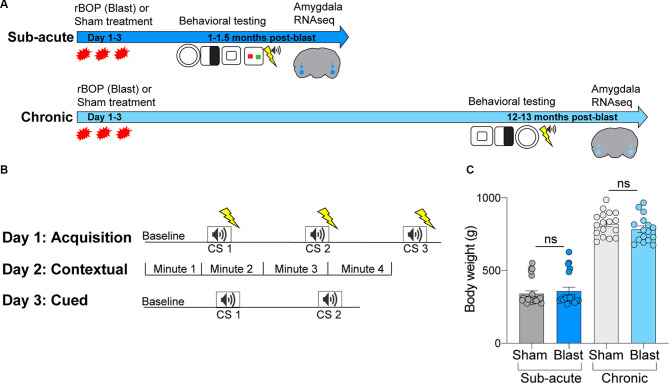
Methodologies for blast exposure and behavioral testing. **(A)** Timeline for blast exposure, behavioral experiments, and tissue collection for the sub-acute and chronic cohorts. **(B)** Schematic of fear conditioning protocols, including acquisition, cued, and contextual fear conditioning. **(C)** Body weights of sub-acute and chronic cohorts at time of sacrifice (ns, not statistically significant; error bars represent SEM).

For the characterization and assessment of behavioral changes that emerge after rBOP exposure, the study employed a condensed form of a previously described behavioral assessment battery that has been shown to possess utility in profiling the multi-faceted behavioral phenotypes associated with blast exposure (Elder et al., 2012). Rats were tested at both a sub-acute (1–1.5 months following rBOP) and chronic (12–14 months following rBOP) timepoints (Figure 1A). The streamlined battery of tests consisted of 4–6 of the nine tasks from the original behavioral assessment battery—including the open field test (OFT), novel object recognition, light/dark box, elevated zero maze (EZM), prepulse inhibition (PPI) and fear conditioning (see cohort-specific behavioral tests in Figure 1A). Note that novel object recognition and PPI were not performed in the chronic cohort because no blast-related effects were found in the acute cohort, and we opted to focus only on the anxiety-related traits for which we had prior robust findings chronically in this paradigm (Elder et al., 2012). Therefore, we do not report findings for novel object recognition or PPI in the current manuscript as they do not directly relate to anxiety-like phenotypes.

#### Open Field Test (OFT)

Locomotor function and anxiety-like behaviors were assessed in the OFT for 10 min for two consecutive days (only Day 1 reported). For the sub-acute cohort, the open field apparatus was a 48 × 48 cm square. Because of the significantly larger size of the aged rats in the chronic cohort (Figure 1C; no differences between treatment groups), we used a larger chamber. The apparatus comprised a white 120 × 120 cm square base and white 60 cm-high walls. The “center” of the open field arena measured 3,600 cm^2^; the center region was determined by dividing the base into 16 identical 30 × 30 cm squares and selecting the four most central squares. Animals were acclimated to the testing room for 1 h before beginning the task. At the start of each trial, subjects were positioned in the corner of the apparatus facing away from the center of the open field. Data were automatically acquired by ANY-Maze software (Stoelting Co., Wood Dale, IL, USA). The apparatus was cleaned before and between trials first with Clidox, then with 70% ethanol.

#### Light/Dark Box

We also used the light/dark box as another measure of anxiety-like behavior. The arena was an opaque black Plexiglas box (40 × 40 cm) enclosing the right half of the interiors so that only the left side was illuminated. Animals were allowed a 60 min habituation period to the chamber on day 1, and on day 2 animals were placed in the dark compartment and allowed to freely explore for 10 min with access to the light side through an open doorway located in the center of the divider. Beam breaks at the ground level were used to detect motion in the dark vs. light side using Versamax activity monitor. We measured the time spent in the light side of the chamber as an index of anxiety-like behavior. The apparatus was cleaned before and between trials first with Clidox, then with 70% ethanol.

#### Elevated Zero Maze (EZM)

To further assess anxiety-like behaviors, subjects were tested on the EZM for 5 min for two consecutive days (only Day 1 reported). The EZM apparatus (San Diego Instruments, San Diego, CA, USA) was a 5.1 cm-wide, black, acrylic, annular platform that measures 61 cm in diameter placed 61 cm above floor level. The platform was equally divided into two pairs of walled (closed) and un-walled (open) quadrants such that the opposite quadrants were identical. Animals were acclimated to the testing room for 1 h before beginning the task. At the start of each trial, subjects were placed in the closed arm closest to the door facing away from the investigator. The number of animals tested for EZM was slightly smaller than other behavioral tasks in the chronic cohort because some animals from both blast and sham conditions were unable to complete this task due to body size (with no group difference in the completion rate). All trials were recorded with a video camera mounted overhead and data were automatically acquired by ANY-Maze. The maze was cleaned before and between trials first with Clidox, then with 70% ethanol.

#### Cued and Contextual Fear Conditioning

Cognitive functions related to associative fear learning were evaluated using cued and contextual fear conditioning as we have described previously (Elder et al., 2012). In brief, the fear conditioning procedure consists of three parts and is conducted over 3 days (Figure 1B). The procedure utilized a rodent conditioning chamber (Coulbourn Instruments, Inc., Lehigh Valley, PA, USA) equipped with a grid floor shocker and positioned within a sound-attenuating cubicle. Our paradigm used a 2-s long foot shock (0.7 mA) as the unconditioned stimulus (US) and a 20-s long, 2 kHz, 80 dB tone as the conditioned stimulus (CS). On day 1, subjects underwent a training trial that paired the conditioned stimulus (CS) and the unconditioned stimulus (US). On day 2, contextual fear conditioning was evaluated by testing subjects in the same environment as the training trial without any presentation of CS and/or US. Finally, on day 3, cued fear conditioning was tested by placing animals in a novel environment with presentations of the CS but not US. Freezing responses were recorded and quantified using Freeze Frame video tracking software (Actimetrics, Coulbourn Instruments, Incorporation).

### Tissue Collection and Processing

Subjects were sacrificed ~1–2 weeks after the last behavioral test by rapid decapitation under light anesthesia (4% isoflurane). Whole brains were extracted and flash frozen in methyl butane chilled with dry ice. Frozen brains were subsequently coronally sectioned using a 1 mm brain matrix. Sections were gently brushed onto slides and stored at −80°C. Bihemispheric tissue samples of the central nucleus/ basolateral (CeA/BLA) amygdala were collected for each subject using 1.50-mm tissue punches and stored for downstream processing. Punches were taken between approximately −2.6 mm and −3.2 mm from Bregma (Campolongo et al., 2009).

### RNA Isolation and Sequencing

Amygdala samples were homogenized in 400 μl of DNA/RNA Lysis Buffer as per the manufacturer’s instructions (Zymo Research Incorporation). Purified total RNA samples were aliquoted and stored at −80°C until submission for RNA sequencing. RNA sequencing libraries were prepared using the KAPA Stranded RNA-Seq Kit with RiboErase (Kapa Biosystems) following the manufacturer’s instructions and sequenced on an Illumina HiSeq2500 sequencer (v4 chemistry) using 2 × 50 bp cycles. The alignment was done with STAR 2.4.2a. STAR index was created using a splice junction annotation database for guidance and both genome sequence and annotation files of Ensembl Rnor 6.0. Quantification of gene expression was achieved through featureCounts 1.4.3-p1 with Ensembl Rnor 6.0 annotation. The RNA-seq data are available on the GEO database under the accession code GSE155393.

### Statistical and Bioinformatics Analyses

#### Behavioral Assessment Analyses

Statistical analyses were performed with Graphpad Prism software. To compare group differences between sham and blast animals in the sub-acute and chronic cohorts, we used unpaired *t*-tests (OFT, EZM, and baseline freezing for fear conditioning). Because the aforementioned statistical tests require normal distribution and equal variance between groups, we performed an F-test of variance and a Kolmogorov-Smirnov test (to test normality) for each dataset and only performed the stated tests if *p* > 0.05 for variance and normality tests. If data was not normally distributed according to the Kolmogorov–Smirnov test, we used the Mann–Whitney test or Wilcoxon test to compare ranks. For fear conditioning data, we used two-way repeated-measures (RM) ANOVAs (acquisition, cued, and contextual fear conditioning) with the Geisser-Greenhouse correction to account for unequal variance and non-normal distribution of data. Statistical significance was denoted at *p* < 0.05, and nominal changes approaching significance were considered trending and denoted at *p* < 0.1.

#### RNA Sequencing Analyses

To analyze transcriptome-wide gene expression changes in rat amygdala, a simple linear model was used to compare different conditions (blast vs. sham exposure) or time (chronic vs. sub-acute time-point) using the voom method (Law et al., 2014) in the Bioconductor (Gentleman et al., 2004) package Limma (Ritchie et al., 2015). We identified differentially expressed genes with a logFC between −1.2 and + 1.2 and with raw or adjusted *p*-values < 0.05 (Figure 4). To assess differences in variability of amygdala gene expression between conditions as well as time, variance ratios were calculated and histograms were generated (Figure 4), and the Kolmogorov-Smirnov test was used to compare two distributions. Furthermore, interaction plots were created to depict the interaction between treatment and timepoint for candidate genes previously implicated in neurodegeneration and neurotrauma (Figure 5).

**Figure 2:**
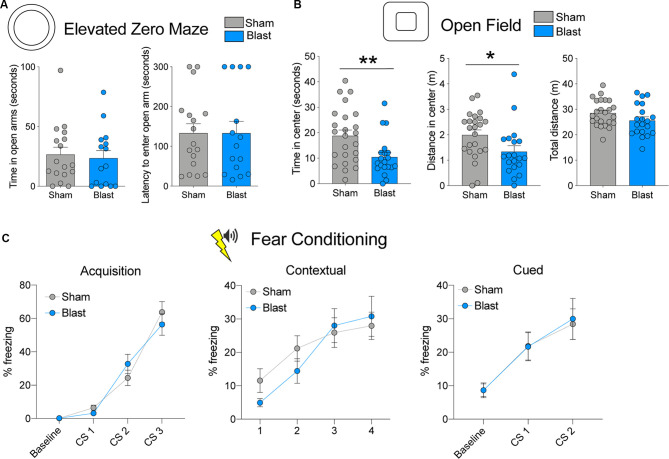
Behavioral testing results for the sub-acute cohort. **(A)** Time spent in open arms and latency to enter open arms for the elevated zero maze (EZM). **(B)** Time spent in the center, distance traveled in the center, and total distance traveled in the open field test (OFT). **(C)** Percent freezing levels during acquisition, contextual fear testing, and cued fear testing (error bars represent SEM; **p < 0.01, *p < 0.05).

**Figure 3:**
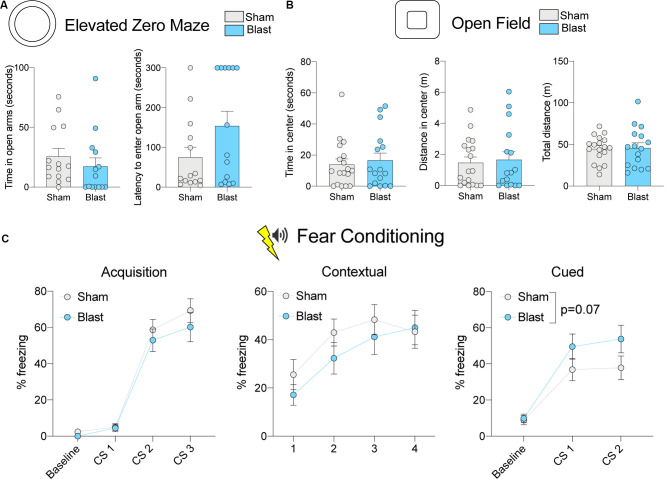
Behavioral testing results for the chronic cohort. **(A)** Time spent in open arms and latency to enter open arms for the EZM. **(B)** Time spent in the center, distance traveled in the center, and total distance traveled in the OFT. **(C)** Percent freezing levels during acquisition, contextual fear testing, and cued fear testing. *P*-value corresponds to the main effect of blast treatment (error bars represent SEM).

**Figure 4:**
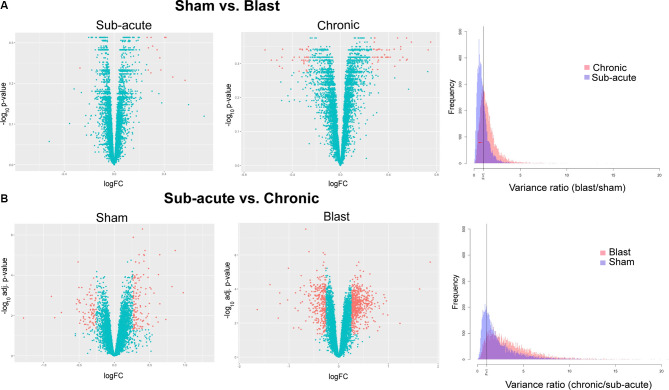
Differentially expressed genes and variance ratios. Volcano plots depicting differentially expressed genes (orange; |logFC| ≥ 1.2 and raw or adjusted *p*-values ≤ 0.05) between **(A)** blast vs. sham animals within the sub-acute and chronic cohorts and **(B)** chronic vs. sub-acute time points with the sham and blast groups. Histograms depicting variance ratios to demonstrate variability within gene expression are also shown.

**Figure 5:**
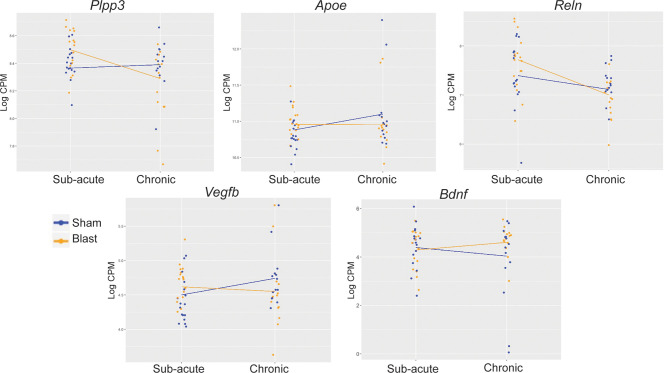
Interaction plots for traumatic brain injury (TBI)- and neurodegeneration-associated candidate gene loci. Interaction analyses identified *Plpp3* as displaying a significant interaction between blast exposure and timepoint after correction for multiple testing (adj. *p*-value < 0.05). Of the other significant interactions (unadjusted *p* < 0.05), we chose to depict interaction plots for TBI- and neurodegeneration-related genes *Apoe, Vegfb, Reln*, and *Bdnf*.

We also used Ingenuity Pathway Analysis (IPA^1^; Qiagen, Inc.) for the identification of canonical pathways and detection of significant gene networks. The causal network analysis identified functional enrichment of gene categories for our datasets, and each category was considered significantly activated (or inhibited) if overlap *p*-value < 0.05 (correcting for multiple testing *via* the Benjamini–Hochberg method (Benjamini and Hochberg, 1995)) and if the activation z-score was between −2 and + 2. Detailed descriptions of IPA analysis are available on the Qiagen website.

## Results

### rBOP Exposure Increases Anxiety-Like Behavior in a Sub-acute Manner but With Diminished Behavioral Effects in the Chronic Cohort

To measure anxiety-like behavior in rats that were exposed to rBOP or sham treatment, we used the EZM (*n* = 17 sham, 15 blast) and OFT (*n* = 24 sham, 20 blast), which are both well-characterized in detecting anxiety-like behavior in rodent models. In the sub-acute cohort of rats, which were tested 1–1.5 months following rBOP or sham treatment, we did not detect changes in anxiety-like behavior in the EZM (time in open arms: *t*_(30)_ = 0.3594, *p* = 0.722, latency: *t*_(30)_ = 0.000, *p* = 0.999; Figure 2A) or the light/dark box (Supplementary Figure S1A, Mann–Whitney *U* = 230.5, *p* = 0.828). In the OFT, however (Figure 2B), blast animals showed increased anxiety compared to sham controls, spending less time in the center (Mann–Whitney *U* = 131, *p* = 0.009) and traveling less distance in the center compared to sham animals (Mann–Whitney *U* = 131, *p* = 0.009), with no significant change in total distance traveled in the arena (*t*_(42)_ = 1.697, *p* = 0.097).

We also tested fear-related cognitive behavior in the same animals using cued and contextual fear conditioning (*n* = 24 sham, 20 blast; Figure 1B; Figure 2C). There were no differences between sham and blast conditions in baseline freezing levels (Mann–Whitney *U* = 230, *p* = 0.776). For the acquisition phase, a two-way repeated-measures ANOVA revealed an effect of CS presentation (*F*_(1.697,71.25)_ = 74.77, *p* < 0.001), showing that both groups were able to learn the association between tone and shock pairing, but there was no effect of blast treatment (*F*_(1,42)_ = 0.037, *P* = 0.855) or CS × blast treatment interaction (*F*_(2,84)_ = 1.635, *p* = 0.201). There were likewise no effects of blast treatment on freezing in contextual (two-way RM ANOVA treatment effect (*F*_(1,42)_ = 0.209, *p* = 0.650) or cued (two-way RM ANOVA treatment effect *F*_(1,42)_ = 0.015, *p* = 0.904) fear conditioning tests (Figure 2C). We ruled out the effects of blast-induced impairments of auditory functioning on fear conditioning due to auditory startle responses being intact during PPI testing for both sham and blast groups (data not shown).

To identify whether the same changes in anxiety-like behavior were present in a chronic manner following rBOP, we performed the same tests on a cohort of subjects that were left undisturbed for 12–13 months following rBOP (Figure 3). Attrition rates did not differ between the blast and sham animals (three sham and four blast animals died before chronic behavioral testing timepoint). In the EZM (*n* = 14 sham, 14 blast; Figure 3A), there was no effect of blast treatment on time in the open arms (Mann–Whitney *U* = 65, *p* = 0.134) or latency to enter an open arm (Mann–Whitney *U* = 70, *p* = 0.206). While there were no significant effects on the EZM, it should be noted that six out of 14 rats (43%) in the blast condition did not enter an open arm during the entire session while only 1 of the 14 sham rats (7%) did not enter an open arm during the session, suggesting a possible increase in anxiety-like behavior in the blast treatment chronic cohort. There were also no significant effects of treatment in the chronic cohort for any measures in the OFT (*n* = 18 sham, 16 blast; Figure 3B), including time in the center (Mann–Whitney *U* = 141, *p* = 0.925), distance traveled in the center (Mann–Whitney *U* = 142.5, *p* = 0.966), and total distance traveled in the arena (*t*_(32)_ = 0.1797, *p* = 0.858). As noted previously in the “Materials and Methods” section, a larger open field chamber was used for the chronic cohort of animals due to a much larger body size after aging, although there were no differences in body weight between blast and sham in the sub-acute (Mann–Whitney *U* = 221, *p* = 0.662) or chronic (*t*_(31)_ = 1.227, *p* = 0.229) cohort at the time of sacrifice (Figure 1C). We also found no effects of blast treatment on time in the center of the light-dark box (Supplementary Figure S1; Mann–Whitney *U* = 101, *p* = 0.195).

We likewise tested fear-related associative learning in the chronic cohort of animals using cued and contextual fear conditioning (*n* = 18 sham, 15 blast; Figure 1B; Figure 3C). During the acquisition phase, blast animals had a lower level of baseline freezing (Mann–Whitney *U* = 64.5, *p* = 0.002). Additionally, during the acquisition phase, a two-way RM ANOVA revealed an effect of CS presentation on freezing (*F*_(1.736,53.81)_ = 78.70, *p* < 0.001), showing that both groups were able to learn the association between tone and shock pairing, but there was no effect of blast treatment (*F*_(1,31)_ = 1.041, *p* = 0.316) or interaction between CS and blast treatment (*F*_(2,62)_ = 0.3486, *p* = 0.707). There were also no significant differences in freezing between sham and blast groups in the contextual fear conditioning test (two-way RM ANOVA treatment effect *F*_(1,31)_ = 0.7681, *p* = 0.388; Figure 3C). In the cued fear conditioning test, blast animals showed a trending increase in freezing upon exposure to the cue that was previously paired with shock (*F*_(1,31)_ = 3.638, *p* = 0.066; Figure 3C).

### Blast Exposure Alters Gene Expression in the Amygdala Both Sub-acutely and Chronically

Due to the increase in anxiety-like behavior for blast animals that were present in a time-dependent manner, we measured gene expression in the amygdala, a brain region highly implicated in anxiety-like behavior, at both sub-acute and chronic time-points in sham and blast animals. We used next-generation sequencing to assess levels of mRNA transcripts in amygdala tissue and data showed a multitude of changes in transcript abundance between groups. First, we compared transcriptomes of blast vs. sham animals within the sub-acute timepoint and found that 14 genes were significantly altered (raw *p* < 0.05) in the blast group compared to sham animals (13 upregulated, 1 downregulated) with none of these changes survived multiple testing correction (Figure 4A; all adjusted *p* > 0.05). Within the chronic time-point, we also found transcriptional changes, with 78 genes significantly altered (raw *p* < 0.05) in the blast group compared to sham (44 upregulated, 34 downregulated), and again none of these comparisons survived multiple testing correction (Figure 4A; all adjusted *p* > 0.05). We further examined the relative variability in gene expression by treatment group. Examining the relative gene expression ratios in blast vs. sham animals within the sub-acute and chronic timepoints respectively, we observed greater variability in gene expression profiles comparing sham and blast in the chronic time point (Figure 4A; Kolmogorov–Smirnov test, *p*-value < 0.001).

As expected due to the typical effects of aging, a large number of genes (215 total) were differentially expressed (adjusted *p* < 0.05) when we compared chronic vs. sub-acute time-points within the sham group (Figure 4B; 129 upregulated, 86 downregulated). Most striking were the effects of time-point within the blast group, where we found 845 genes differentially expressed (adjusted *p* < 0.05) in the chronic vs. sub-acute time-point within the blast group (Figure 4B; 562 upregulated, 283 downregulated). To capture the relative variability associated with blast-exposure longitudinally, we examined the variability in gene expression in chronic vs. sub-acute time points in blast and sham groups separately. Specifically, we computed the variance ratios in the chronic vs. sub-acute animals in each treatment group and found that the distribution of the variance ratios was significantly different between the blast and sham groups with the blast group showing greater variability (Figure 4B; Kolmogorov–Smirnov test, *p*-value < 0.001).

### Gene Expression Changes Associated With Chronicity of Blast Exposure

While we found changes between sham and blast groups within both the sub-acute and chronic time points, the most significant change was the abundance of genes differentially expressed within the blast group when we compared chronic vs. sub-acute time points, suggesting an interaction between treatment and chronicity of exposure post-blast. In an exploratory analysis, we examined the interaction between treatment (blast vs. sham) by time-point (chronic vs. sub-acute). The *Plpp3* gene showed significant interaction for treatment by time effect (*p* = 0.007 adjusted for multiple testing). In addition to *Plpp3*, we also identified other loci that have been previously implicated in neurotrauma and neurodegeneration, including *Apoe, Reln, Vegfb*, and *Bdnf*, which likewise show interaction effects in this model (raw *p* < 0.05; Figure 5).

### Gene Networks Associated With Chronicity of Blast Exposure

**Figure 6:**
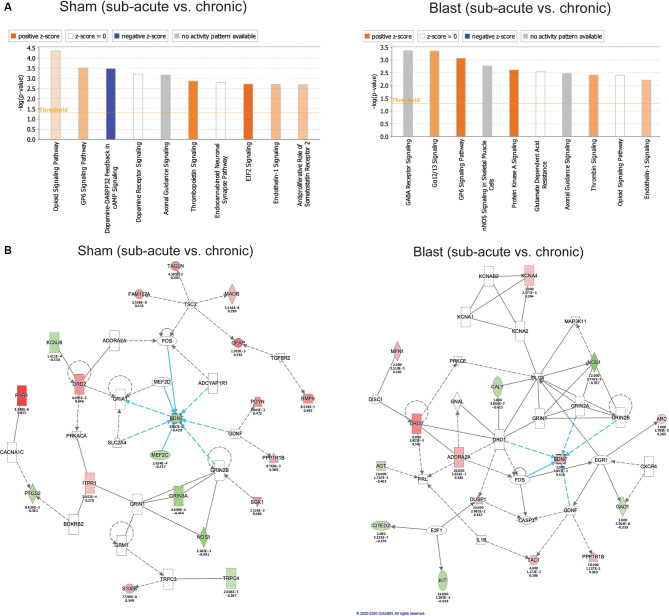
Gene ontology enrichment using Ingenuity Pathway Analysis (IPA). **(A)** Top 10 gene categories. The y-axis gives the negative logarithm function of overlap *p*-value. Orange or blue bar represents a positive or negative activation z-score respectively, as calculated by IPA based on expected directionality of gene expression change within the gene category using the IPA knowledge base. White bars represent a z-score of 0 and gray bars signify that IPA was unable to calculate an activation z-score based on the unavailability of data in their knowledge base for that gene category. **(B)** Top gene network identified by IPA in the sham and blast cohorts comparing chronic vs. sub-acute time points. Green nodes within the network represent downregulated genes in our dataset and red nodes represent upregulated genes in our dataset. Nodes with no color represent IPA-generated genes using the IPA knowledge base. Blue arrows represent pathways interacting directly with *Bdnf* as a hub gene.

We also used Ingenuity Pathway Analysis (IPA; Qiagen, Inc.) to investigate enrichment of genes in known biological pathways using the differentially expressed genes that showed significant fold changes (adjusted *p* < 0.05) in the chronic vs. sub-acute time points within each of the treatment groups (blast and sham respectively; Figure 4B). We first compared genes differentially expressed in the chronic vs. sub-acute time points within the sham group, which should represent normal aging processes. Of the top 10 canonical pathways enriched in our dataset (Figure 6A), the majority were enriched for genes that code for enzymes and ion channels for opioid, GP6, and dopamine signaling. We conducted the same assessment for the chronic vs. sub-acute timepoint within the blast group and found an altered set of canonical pathways (Figure 6A), with the top hit being the GABA receptor signaling pathway, a pathway that was not enriched significantly (*p* > 0.05) in the sham dataset. This gene category included *Adcy1, Adcy8, Cacna1d, Cacna*1 h*, Cacng4, Gabrg1, Gad1, Gad2, Gnas, Kcnh2, Slc32a1, Slc6a1, Slc6a11*.

Because of the altered set of functionally enriched genes between our sham and blast groups when comparing time points, we used a Comparison Analysis on IPA to compare the gene networks that are significantly altered in the sham (chronic vs. sub-acute) dataset and the blast (chronic vs. sub-acute) dataset. We identified the top gene network for each condition (blast and sham) and both were enriched for genes involved in neurological disorders and organismal injury and showed a large overlap between the blast and sham conditions (Figure 6B). The most notable difference between these conditions was an oppositional effect on *Bdnf*, which is the hub of this gene network, and we had previously identified this as an important gene showing the interaction of treatment and age.

## Discussion

The short and long-term neurological and psychological consequences of mTBI are of immense interest to the Veteran community. In the current study, we aimed to further define the behavioral and molecular effects of blast-related mTBI using a well-established rBOP model in rats. We exposed adult male rats to the rBOP procedure and conducted behavioral tests for anxiety and fear conditioning at 1–1.5 months (sub-acute) or 12–13 months (chronic) following blast exposure.

In the cohort of animals tested at the sub-acute timepoint, there was a robust increase in anxiety-like behavior in the OFT (although no changes detected in the EZM), but no change in acquisition of fear learning or cued and contextual fear memory. In the chronic cohort, blast animals showed only a trending increase in anxiety-like behavior in the EZM and no change in fear memory acquisition, but a trending increase in freezing to a tone CS after cued fear conditioning. An increase in anxiety-like behavior has been shown in multiple rodent models of TBI (Meyer et al., 2012; Almeida-Suhett et al., 2014; Heldt et al., 2014; Davies et al., 2016; Zuckerman et al., 2016), including our own (Elder et al., 2012), and our current data are in line with these findings. As discussed previously, the majority of studies in the field measure anxiety at a relatively short time interval post-blast, and our sub-acute timepoint likewise shows a significant increase in anxiety 1.5–2 months post-blast. Conversely, we did not find a statistically significant change in anxiety-like behavior 12–13 months post-blast (chronic timepoint), which is in concordance with recent work showing that over time the anxiety-like phenotype is diminished over time, as shown by the EZM and other anxiety behavior tests (Popovitz et al., 2019). We also measured the acquisition of fear learning and both cued and contextual fear and found a trending effect of rBOP at chronic timepoint but no sub-acute effects. Some human studies (Glenn et al., 2017) and rodent models of the blast (Meyer et al., 2012; Davies et al., 2016) have found impaired fear acquisition following blast exposure, but there are still mixed findings of the effects of blast on cued and contextual fear recall (Elder et al., 2012; Meyer et al., 2012; Teutsch et al., 2018).

While an anxiety phenotype was not detected at the chronic time-point in our model, previous rodent studies from our lab (Perez-Garcia et al., 2016; Gama Sosa et al., 2019) and human studies (Glenn et al., 2017) suggest that long-term pathology exists following blast exposure that may contribute to the lasting deleterious effects of blast in the Veteran population. We chose to measure gene expression in the amygdala of rats that were exposed to rBOP because of the importance of this brain region in mTBI-related psychopathology, including anxiety and PTSD. An abundance of evidence exists that amygdala connectivity and structure is altered following exposure to TBI, leading to aberrant anxiety-like behavior (Han et al., 2015; Palmer et al., 2016; Tate et al., 2016; Popovitz et al., 2019). Changes in mRNA and protein levels for specific gene loci have also been identified in the rodent amygdala following TBI, including *Thy1* (Heldt et al., 2014), *Stathmin1* (Elder et al., 2012), and GABA-related proteins (Almeida-Suhett et al., 2014). We used a transcriptome-wide, unbiased approach to measure gene expression in the central/basolateral amygdala at a sub-acute and chronic time-point following rBOP. Within the sub-acute and chronic time points, we compared sham and blast conditions and found no differentially expressed genes that passed correction for multiple testing. Among the differentially expressed genes that had a *p*-value of <0.05 before correction, the chronic timepoint had a much larger abundance of differences, suggesting the amygdalar transcriptome is susceptible to more drastic changes after a significant delay post-injury.

Most notably we identified a strong interaction between blast condition and age. In comparison to the subtle effects between sham and blast at each time-point, when we compared the transcriptome of sham animals at the chronic vs. sub-acute time-points, we found an abundance of genes differentially expressed and surviving correction for multiple testing. These changes emphasize the large effect of aging on gene expression in the amygdala, and we identified significant alterations in expression levels of various genes that have previously been associated with normal aging and neurodegeneration, including *Gfap* (Rodríguez et al., 2014), *Vegfb* (Mahoney et al., 2019), and *Bdnf* (Tapia-Arancibia et al., 2008; Guilloux et al., 2012). When we compared the chronic vs. sub-acute transcriptomes within the blast group, however, we identified the greatest number of differentially expressed genes, exemplifying the treatment by age interaction after mTBI, which we confirmed *via* interaction analyses. Significant interactions included *Plpp3*, which is enriched in the ventral midbrain and has been linked to dopaminergic functioning in the striatum but not yet linked to the amygdala function (Gómez-López et al., 2016). Of the abundant interactions that did not survive multiple testing correction, we chose to highlight four additional genes that have known roles in brain injury and/or amygdala functioning, including *Apoe* (Klein et al., 2014; Tang et al., 2015), *Reln* (Boyle et al., 2011; Frankowski et al., 2019), *Vegfb* (Wu et al., 2008; Savalli et al., 2014; Mahoney et al., 2019), and *Bdnf* (Wu et al., 2008; Guilloux et al., 2012; Sagarkar et al., 2017), which are all crucial mediators of synaptic plasticity and neurotransmission as well as structure and function of brain vasculature. Interestingly, *Plpp3*, *Apoe*, *Reln*, and *Vegfb* show similar patterns of interaction, such that expression levels in blast animals are higher than sham animals at the sub-acute timepoint but lower than sham animals at the chronic time point. *Bdnf* however shows the opposite pattern, such that blast animals have lower *Bdnf* expression compared to shams at the sub-acute timepoint, but higher *Bdnf* expression compared to shams at the chronic time point.

Additionally, as evidenced by IPA analyses of canonical pathways enriched in both datasets, the differentially expressed genes in the aging amygdala within the blast group are enriched for GABA signaling pathways (including *Adcy1, Adcy8, Cacna1d, Cacna*1 h*, Cacng4, Gabrg1, Gad1, Gad2, Gnas, Kcnh2, Slc32a1, Slc6a1, Slc6a11*), while this pathway is absent from the sham group. Impaired GABA signaling in the amygdala following TBI has been established, including loss of GABAergic interneurons and decreased frequency of GABA receptor-mediated inhibitory postsynaptic currents (Almeida-Suhett et al., 2014). This decrease in inhibition leading to increased neuronal excitability in the amygdala may be a crucial mediator of anxiety-like behavior after brain injury. Previous work shows that decreased GABAergic functioning in the amygdala can directly lead to altered performance on the OFT, EPM, and fear conditioning (Babaev et al., 2018). Further, *Adcy8* codes for a protein kinase that has been repeatedly implicated in anxiety-like behavior, synaptic plasticity, and mood disorders (Schaefer et al., 2000; de Mooij-van Malsen et al., 2009; Bernabucci and Zhuo, 2016; Tanaka et al., 2019). A recent study found changes in anxiety-like behavior linked to a rodent PTSD-like model accompanied by aberrant *Adcy8* expression in the amygdala (Tanaka et al., 2019). Similar to our results, the stress-induced change in *Adcy8* expression was dependent on a time delay following the stressor, highlighting the importance of the treatment by timepoint interaction in regulating brain function and behavior after a stressor.

When we used IPA to identify gene networks involved in the interaction between blast exposure and timepoint, the top networks involved *Bdnf*, which we had already established displayed a statistically significant interaction (Figure 5). *Bdnf* codes for a protein that is crucial for neural plasticity and development and has been implicated widely in psychiatric disorders (Zagrebelsky and Korte, 2014). There is evidence in post-mortem human brain tissue from depressed subjects that *Bdnf* expression may correspond to GABA-related dysfunction in the amygdala (Guilloux et al., 2012), similar to our findings in aged mTBI rats. Additionally, many groups have demonstrated the importance of *Bdnf* expression following TBI in humans (Rostami et al., 2011; Failla et al., 2014; Korley et al., 2015) and animals (Griesbach et al., 2002; Wu et al., 2004, 2008; Feliciano et al., 2014) and have suggested alterations in *Bdnf* *via* pharmacology or experience as a potential therapeutic target following TBI (Griesbach et al., 2009; Wurzelmann et al., 2017). One group found that *Bdnf* levels were decreased after a short time interval post-TBI (Sagarkar et al., 2017), but did not investigate a later timepoint. Our findings here suggest that *Bdnf*, a known regulator of GABA-ergic transmission in the amygdala, may play a role in the aging-related phenotypic outcomes after blast exposure.

A potential limitation of the current study was the use of the same cohorts of animals for both behavioral and molecular assays, which may introduce the stress of behavioral testing as a confounding factor in transcriptomic results, especially when combined with aging and injury. We note however that all animals in both treatment groups (blast and sham) experienced the same testing procedures and therefore experienced the same levels of stress and conditions before molecular assays were performed, addressing this potential confound. Additionally, having additional groups (i.e., sub-acute and chronic × blast and sham) in parallel that did not undergo the behavioral battery increases the scope and costliness of the study, though certainly can be considered in future studies with the availability of greater funds. Another limitation of the current study was the lack of identification of mechanisms regulating gene expression. Exact mechanisms for gene expression regulation after blast exposure are still unknown, but DNA methylation and other epigenetic mechanisms may play a role in these outcomes. The *Bdnf* gene locus is heavily modified by epigenetic machinery which contributes to the experience- and activity-dependent nature of *Bdnf* expression (Boulle et al., 2012). We previously found changes in DNA methylation in the cortex after our rodent model of mTBI sub-acutely (Haghighi et al., 2015), and future studies will investigate DNA methylation at the chronic time point, which will likely uncover mechanistic insights into the abundant changes in gene expression we found here. Additionally, the current study focused solely on mRNA levels following blast exposure that may contribute to behavioral phenotypes, but in future investigations, we will identify protein levels for specific genes of interest to better link transcriptomics to behavioral outcomes. Future studies will also link gene expression in our animal models to changes observed in human cohorts using peripheral measures such as blood and saliva to allow a more translational approach.

Overall, the current study showed evidence for anxiety-like behavioral effects that may be related to amygdalar transcriptomic changes following blast exposure, and these changes occur in an age-dependent manner. Gene networks that we identified could provide crucial insights into the molecular underpinnings of mTBI and provide a link between experience and psychopathology resulting from blast exposure. Understanding the molecular circuitry involved in the long-term psychopathological outcomes resulting from mTBI is critically important to the health and productivity of our Military and Veteran population and will contribute to the development of targeted treatment interventions.

## Data Availability Statement

The original contributions presented in the study are publicly available. This data can be found here: GSE155393.

## Ethics Statement

The animal study was reviewed and approved by Institutional Animal Care and Use Committees of the Naval Medical Research Center and the James J. Peters Veterans Administration (VA) Medical Center.

## Author Contributions

JB and FH wrote the manuscript. AT and SA generated blast-exposed animals. JB, IC, MU, NM, GE, and FH designed and performed animal behavior and animal molecular experiments. JB analyzed the behavioral data and performed IPA analyses on RNA seq data. ZW, YG, and FH analyzed RNA-seq data. All authors contributed to the article and approved the submitted version.

## Conflict of Interest

The authors declare that the research was conducted in the absence of any commercial or financial relationships that could be construed as a potential conflict of interest.

## Footnotes

^1^www.ingenuity.com

## Supplementary Material

FIGURE S1 Additional behavioral testing results. **(A)** Time spent in the light side of the light/dark box in the sub-acute cohort. **(B)** Time spent in the light side of the light/dark box in the chronic cohort (error bars represent SEM).
