# Supplementary figures and images for: Blast-Related Mild TBI Alters Anxiety-Like Behavior and Transcriptional Signatures in the Rat Amygdala
Source: Front Behav Neurosci. 2020 Sep 30;14:160. doi: 10.3389/fnbeh.2020.00160 (PMC7604767; doi:10.3389/fnbeh.2020.00160)

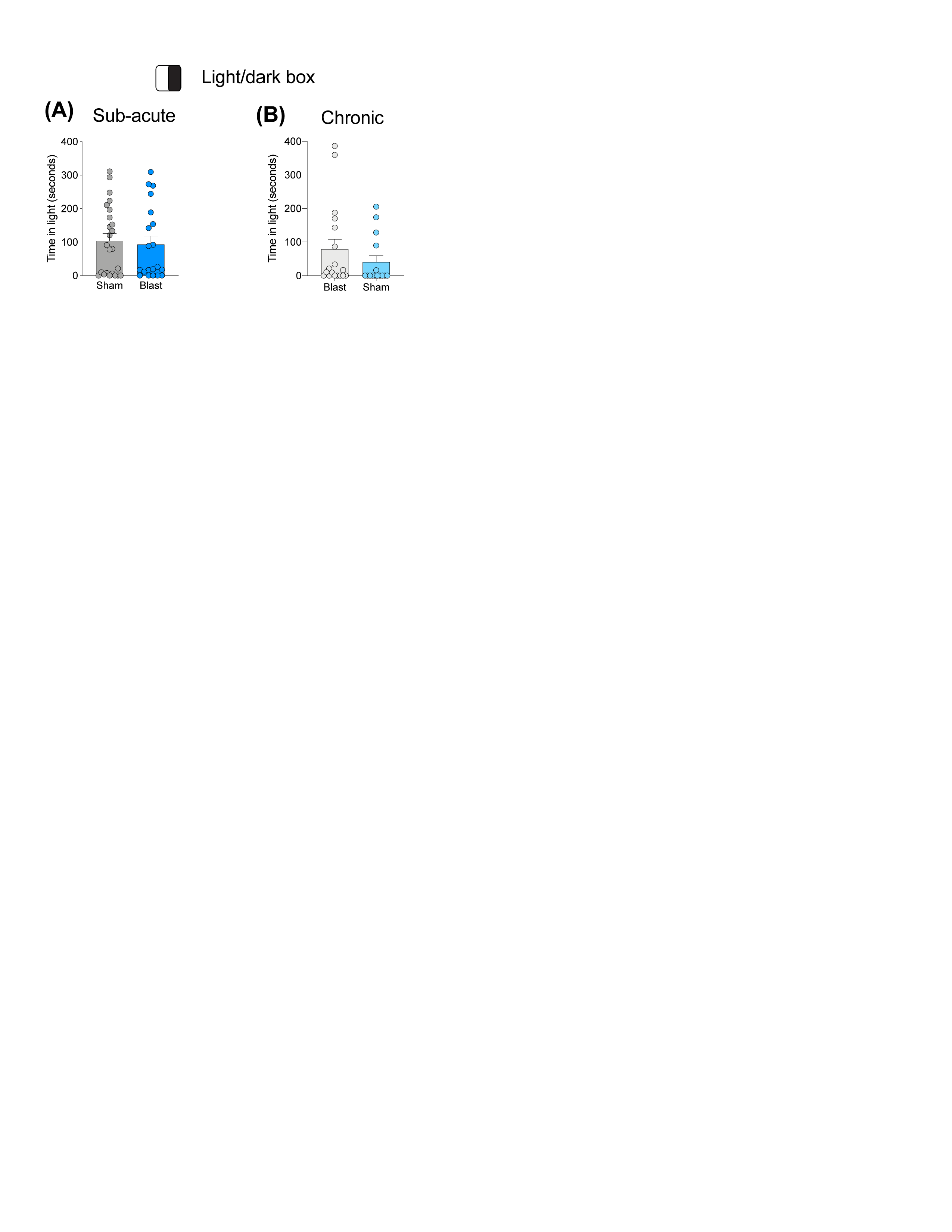

Supplement: FIGURE S1 — Additional behavioral testing results. (A) Time spent in the light side of the light/dark box in the sub-acute cohort. (B) Time spent in the light side of the light/dark box in the chronic cohort (error bars represent SEM). [file Image_1.TIF]
